# Subarachnoid hemorrhage and COVID-19: Association or coincidence?

**DOI:** 10.1097/MD.0000000000023862

**Published:** 2020-12-18

**Authors:** Auricelio Batista Cezar-Junior, Igor Vilela Faquini, José Laercio Junior Silva, Eduardo Vieira de Carvalho Junior, Luiz Euripedes Almondes Santana Lemos, João Batista Monte Freire Filho, Hetevaldo Tavares de Lira Filho, Erton Cesar de Albuquerque Pontes, Nivaldo Sena Almeida, Hildo Rocha Cirne Azevedo-Filho

**Affiliations:** Hospital da Restauração, Department of Neurosurgery, Recife, Av. Gov. Agamenon Magalhães, s/n - Derby, Recife - PE, 52171-011, Brazil.

**Keywords:** coronavirus, COVID-19, pandemic, SARS-CoV-2, subarachnoid hemorrhage

## Abstract

Some evidences suggest the involvement of the central nervous system in patients infected with SARS-CoV-2. We aim to analyze possible associations between coronavirus disease 2019 (COVID-19) pandemic and spontaneous subarachnoid hemorrhage (SAH), in a comprehensive neurological center.

We conducted a retrospective case series of 4 patients infected by COVID-19, who developed spontaneous SAH. Clinical data were extracted from electronic medical records.

Between March 24, 2020, and May 22, 2020, 4 cases (3 females; 1 male) of SAH were identified in patients infected with SARS-CoV-2, in a comprehensive neurological center in Brazil. The median age was 55.25 years (range 36 -71). COVID-19-related pneumonia was severe in 3 out of 4 cases, and all patients required critical care support during hospitalization. The patients developed Fisher grade III and IV SAH. Digital subtraction angiography (DSA) was performed in 3 of the 4 patients. However, in only 1 case, an aneurysm was identified. Inflammatory blood tests were elevated in all cases, with an average D-dimer of 2336 μg/L and mean C-reactive protein (CRP) of 3835 mg/dl The outcome was poor in the majority of the patients, with 1 death (25%); 2 (50%) remained severely neurologically affected (mRS:4); and 1 (25%) had slight disability (mRS:2).

This study shows a series of 4 rare cases of SHA associated with COVID-19. The possible mechanisms underlying the involvement of SARSCoV-2 and SHA is yet to be fully understood. Therefore, SHA should be included in severe neurological manifestations in patients infected by this virus.

## 1 Introduction

In December 2019, the virus named SARS-CoV-2 was identified in Wuhan, China, after an outbreak of unexplained pneumonia cases. In February 2020, this disease was defined by the World Health Organization (WHO) as coronavirus disease 2019 (COVID-19).^[1–3]^ Since the outbreak of the epidemic in Wuhan, China,^[4,5]^ it has spread rapidly across the world, with more than 6.931.000 confirmed cases and 400.850 deaths worldwide to date.^[6]^ It is known that clinical manifestations of SARS-CoV-2 have shown a wide spectrum, ranging from asymptomatic infections, mild respiratory tract disease and severe pneumonia with respiratory failure and associated risk of death.^[7]^ Several studies have described some of the most common symptoms of COVID-19 infection, including fever, cough, diarrhea, fatigue and predominantly respiratory symptoms.^[8]^

However, observational studies have suggested the involvement of the central nervous system (CNS) in patients infected with SARS-CoV-2. Neurological symptoms have been already reported, such as headache, dizziness, hypogeusia, hyposmia. More severe neurological conditions are also observed, such as stroke and vascular events, impairment of consciousness or encephalopathy, seizures, Guillain-Barré syndrome and peripheral nerve disorders.^[9–13]^ However, it is unclear whether SARS-CoV-2 is primarily neurotropic and responsible for neurological damage, or whether these symptoms are attributed to secondary mechanisms.^[12,13,14]^ In addition, intracranial hemorrhages have rarely been reported to date. In this study, we aim to describe the clinical aspects as well as the treatment and the outcomes of 4 rare cases of acute spontaneous subarachnoid hemorrhage (SAH), in patients infected with COVID-19. In 1 case, there was an association with inter-hemispheric acute subdural hemorrhage (IHSDH)

Therefore, in the light of our knowledge, this is the largest case series to date of spontaneous SAH related to SARS-CoV-2. Finally, a question that arises is: according to the recent pandemic by COVID 19, would SHA in that context be an association or coincidence?

## 2 Materials and methods

We conducted a retrospective case series of 4 patients who developed sudden spontaneous SAH during SARS-CoV-2 infection, from a comprehensive neurological center, in Recife, Brazil. It was approved by the local ethics committee of Hospital da Restauração, Recife-Brazil. The patients or family members provided informed consent before participation, in accordance with the Declaration of Helsinki.

Clinical data were extracted from electronic medical records, and neurological data were checked in detail by a team of trained neurosurgeons and neuroradiologists.

The diagnosis of COVID-19 was confirmed by reverse-transcriptase polymerase chain reaction (RT-PCR) from the upper and eventually lower respiratory tract (collected sputum and endotracheal aspirates), according to the recommendations of the WHO.^[14]^ These swab samples were collected and placed into a tube containing preservation solution for the virus. SAH diagnosis was performed by cranial CT-scan (measured using the Fisher scale^[4]^), associated with digital subtraction angiography (DSA), in most cases. In addition, laboratory tests such as coagulation tests, inflammatory markers (C-reactive protein [CRP], LDH and D-dimer) were also performed. Patient outcomes were assessed using the modified Rankin Scale (mRS)^[15]^ at discharge.

## 3 Illustrative cases

### 3.1 Case 1

A 36-year-old woman presented with nausea, cough, fever, anosmia and mild headache for 6 days. There were no reports of comorbidities or previous neurological events. Upon admission to the emergency room, the patient was alert and oriented. His vital signs were stable, and a neurological examination revealed a mild headache and neck stiffness. Body temperature at admission was 37.8°C. Intravenous (IV) antibiotic (ceftriaxone 2 g IV) and antiviral (acyclovir IV) was administered, considering central nervous system (CNS) infection. Due to the report of cough, fever and anosmia in the last 6 days, the hypothesis of infection by the novel coronavirus was raised. Therefore, throat swab samples were collected for 2019-nCoVRNA RT-PCR, which confirmed infection by SARS-CoV-2. Initial laboratory tests showed increased CRP (40 mg/dl, n.v <10), LDH (507 IU/L, n.v. 125–220) and D-dimer (4000 μg/L, n.v. <500). Coagulation tests were normal. CSF analysis showed a pleocytosis (100 cells, n.v. <5 cells/μl) with a predominance of polymorphonuclear cells (60%), and a high red blood cell count (1200, n.v. <10 mm^3^).

**Figure 1:**
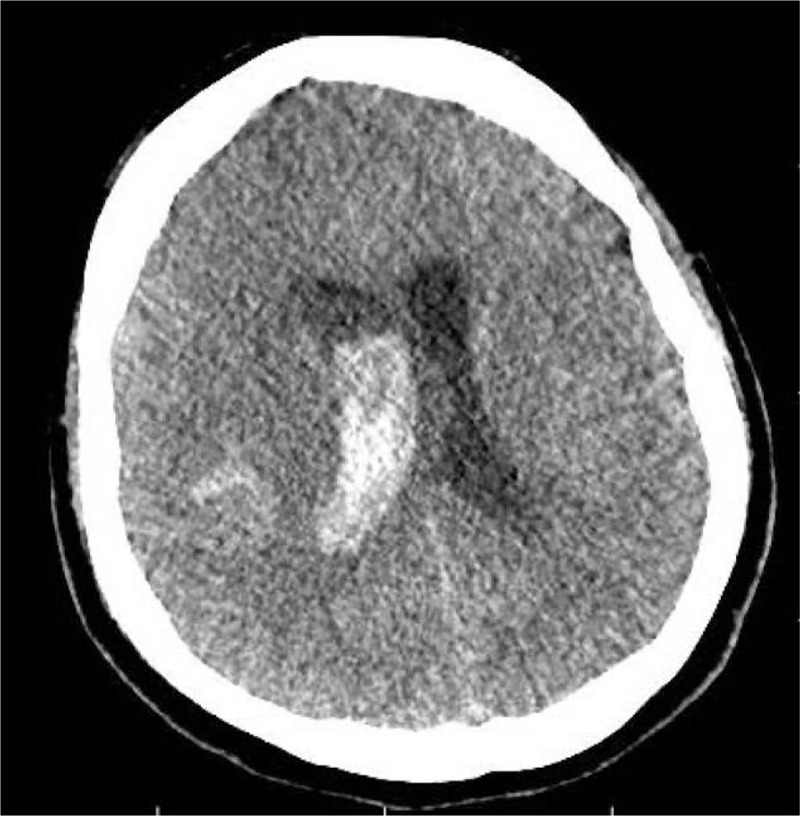
Brain CT-scan showing SAH associated with ventricular hemorrhage.

**Figure 2:**
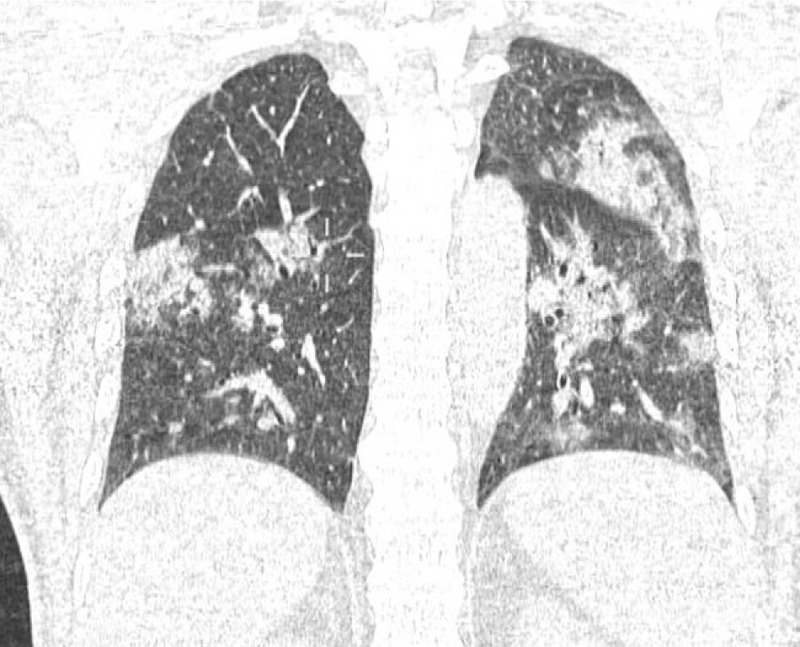
Chest CT-scan in coronal cut showing ground-glass opacities suggestive of pulmonary infection by the novel coronavirus.

**Figure 3:**
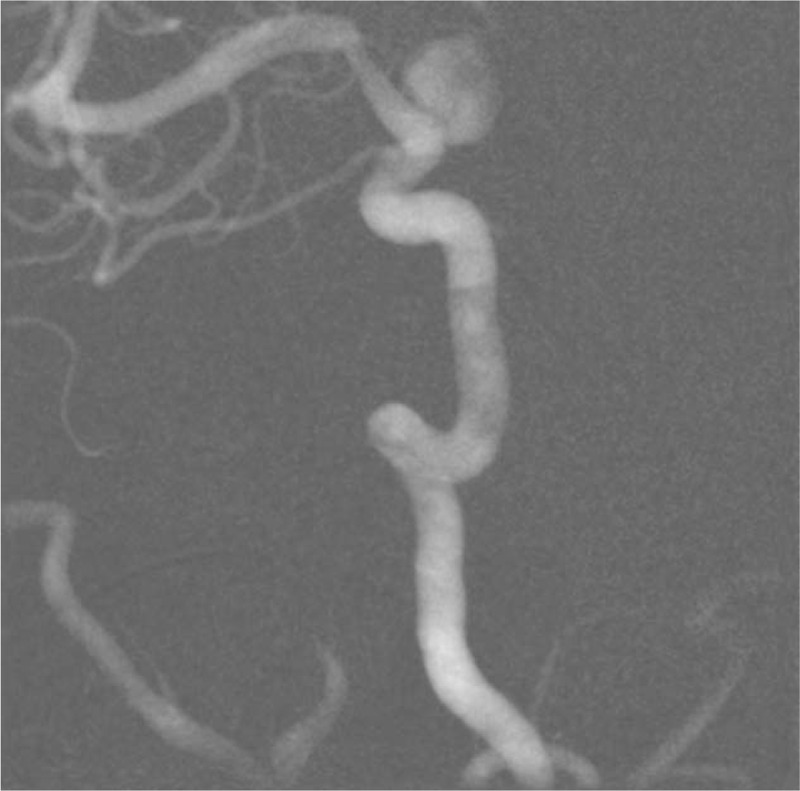
Oblique (B) view of right internal carotid artery angiography, with a saccular aneurysm of its communicating segment.

**Figure 4:**
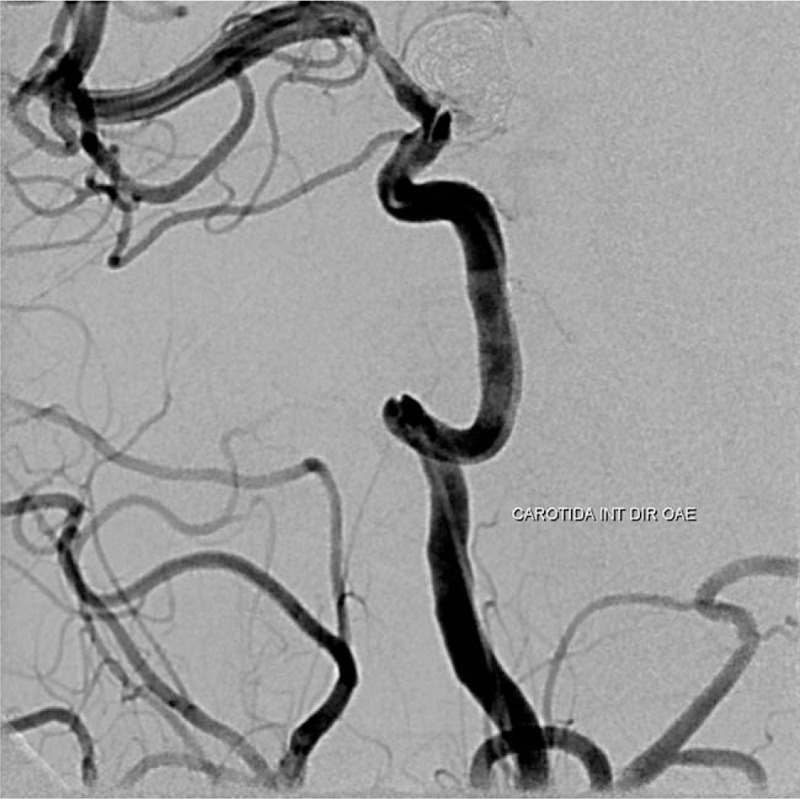
DSA following coil embolization shows the complete aneurysm obliteration.

Within 24 hours, the patient had conscious impairment, requiring mechanical ventilation. The patient was moved to our neurological center and a cranial CT-scan was performed. It showed Fisher grade IV SAH and incipient hydrocephalus were visualized (Fig. 1). Therefore, EVD (external ventricular drain) implantation was performed. Chest CT-scan demonstrated bilateral ground-glass opacities, suggestive of COVID-19 pneumonia (Fig. 2). DSA revealed a ruptured saccular aneurysm in communicating segment of right internal carotid artery (ICA), as shown in Figure 3. No signs of angiographic vasospasm were observed. Balloon-assisted embolization was performed and complete aneurysm occlusion was obtained (Fig. 4). Patient was discharged for rehabilitation after 28 days, with moderate-severe disability (mRS: 4).

### 3.2 Case 2

**Figure 5:**
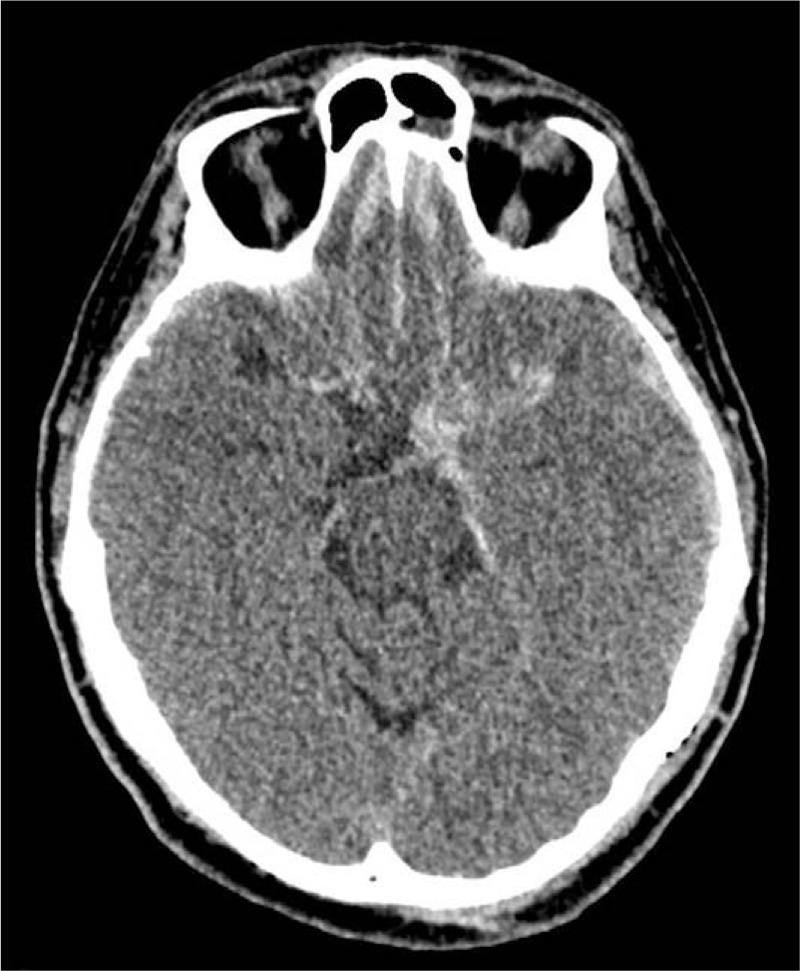
Cranial CT-scan (axial slice) showing SAH occupying the basal cisterns and left Sylvian fissure.

A 53-year-old man presented with a sudden-onset headache for 24 hours. He had a report of fever, cough and dyspnea in the last 4 days. Body temperature at admission was 37.6°C. On examination, he was confused, but had no neurological deficits. Clinical and laboratory findings indicated moderate respiratory distress (PaO2 /FiO2 238). Inflammatory blood markers were high (CRP 30, 4 mg/dl, n.v <10; LDH 344 IU/L, 125–220; D-dimer 1134 ng/ml n.v. <500). Coagulation tests were normal. Chest CT-scan demonstrated ground-glass opacities in more than 50% of the pulmonary parenchyma bilaterally. Brain CT-scan showed Fisher III SAH (Fig. 5). RT-PCR for 2019-nCoVRNA confirmed SARS-CoV-2 infection. Digital angiography was performed within 24 hours, with no evidence of cerebral aneurysms or arteriovenous malformations (AVM). The patient stayed in the ICU for 7 days, without requiring mechanical ventilation. Patient was discharged home after 3 weeks, with good functional status (mRS = 2).

### 3.3 Case 3

**Figure 6:**
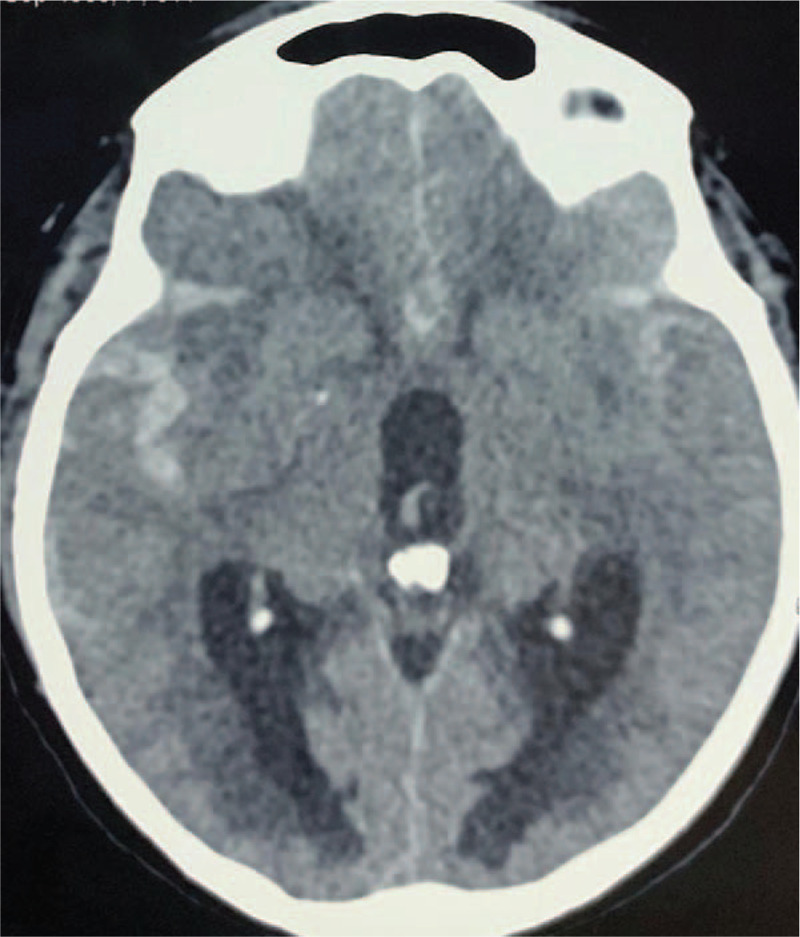
Cranial CT scan (axial slice) shows foci of SAH in both Sylvian fissures (more marked on the right side) and acute hydrocephalus.

A 61-year-old woman presented with cough, fever, and myalgia for 14 days. On the 15th day, she developed dyspnea and pleuritic pain. Clinical and laboratory evaluation showed moderate respiratory distress (PaO2 /FiO2 191). Chest CT-scan it was suggestive of COVID-19. Laboratorial tests showed increased CRP (56 mg/dl, n.v. <10) and D-dimer (2850 μg/L, n.v. <500). Coagulation tests were normal. On the 7th day of ICU stay (23 days after the onset of respiratory symptoms), the patient presented impairment of consciousness, requiring endotracheal intubation. Cranial CT-scan was performed and showed Fisher grade IV SAH, requiring EVD (Fig. 6). Within the first 24 hours, he developed progressive hemodynamic instability and death. Therefore, DSA could not be performed in this case.

### 3.4 Case 4

**Figure 7:**
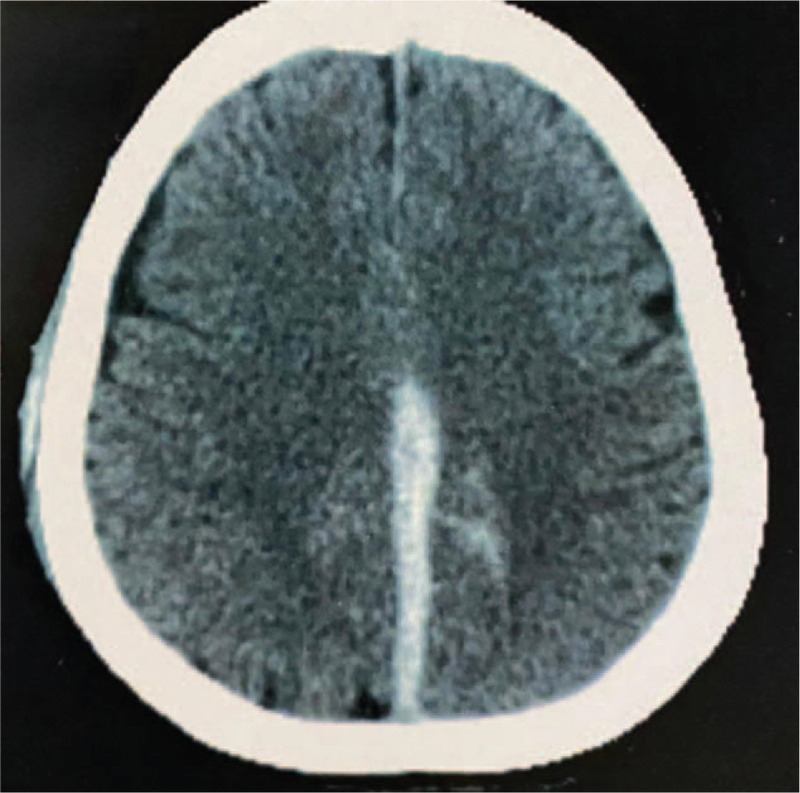
Axial CT-scans showing an acute IHSH associated with SAH (Fisher III), in a 71-year-old patient with confirmed COVID-19.

A 71-year-old woman presented with sudden severe headache associated with right hemiparesis and aphasia for 6 hours. She had fever, cough and myalgia for 7 days. During initial medical care, she had a seizure and a progressive respiratory distress (PaO2 /FiO2: 165), requiring mechanical ventilation. After clinical stabilization, brain and chest CT-scan were performed, showing Fisher III SAH (Fig. 7) associated with Acute Interhemispheric Subdural Hemorrhage (IHSDH), and ground glass affecting more than 50% of the pulmonary parenchyma, respectively. Laboratory tests showed elevated inflammatory markers (CRP 270 mg/dl, n.v. <10; D-dimer 1360 ng/ml, n.v. 0–500). Coagulation tests were normal. DSA was performed and showed no aneurysms, AVMs or abnormalities in the cerebral venous system. Throat swab samples were collected for 2019-nCoVRNA RT-PCR and confirmed SARS-CoV-2 infection. The respiratory condition was treated with Ceftriaxone, Azithromycin and Oseltamivir. New angiogram performed within 3 weeks, remained without new findings. The patient was discharged to rehabilitation (mRS: 4) after 3 weeks of hospitalization, with complete resorption of cerebral hemorrhage in the radiological control.

## 4 Results

Four patients were selected for the study, mostly women (3 females; 1 male). The median age was 55 to 25 years (range 36 −71). COVID-19-related pneumonia was severe in 3 out of 4 cases. All patients required critical care support during hospitalization and showed impairment of consciousness associated with SAH. The most common finding on cranial CT-scan was Fisher IV (50%) and Fisher III (50%) SAH, and one case of IHSDH. Two patients developed hydrocephalus requiring EVD implantation. The mean interval between the onset of SARS-CoV-2 symptoms and SAH was 875 days. Three patients underwent DSA, but cerebral aneurysm was seen in only 1 case. One patient was unable to undergo cerebral angiography due to severe clinical condition, followed by death. Thus, a berry aneurysm rupture was identified and treated with coiling. Ground-glass opacities in chest CT-scan were multiple and bilateral in all cases. Inflammatory markers were quite elevated, with an average D-dimer of 2336 μg/L (range 1420–4000) and CPR of 38,35 mg/dl (range 27 −56). Coagulation tests were normal. The outcome was poor in the majority of the patients: 1 died (25%), 2 (50%) remained severely neurologically affected (mRS: 4) and 1 (25%) had slight disability (mRS = 2). Clinical, radiological and laboratory data for all patients are summarized in Table 1.

**Table 1:** Clinical and neurological presentations in patients with laboratory-confirmed SARS-CoV-2 who experienced SAH.

	Case 1	Case 2	Case 3	Case 4
Sex/age, years	Female/36	Male/53	Female/61	Female/71
Initial symptoms	Nausea, vomiting, fever, anosmia, and headache for 6 days	Fever, cough, and dyspnea for 4 days	Cough, fever, tiredness, and myalgia for 14 days	Fever, cough, anosmia, and myiagia for 7 days.
Respiratory support	Mechanical ventilation	3 L/minutes nasal oxygen	Mechanical ventilation	Mechanical ventilation
Comorbidities	SAH^∗∗^	No	No	SAH^∗∗^, Obesity
Antibiotics used before onset of neurological symptoms	Ceftriaxone, Vancomicin, Meropenem	Oseltamivir	Ceftazidime, Vancomicin, Meropenem	Ceftriaxone, Azithromycin, Meropenem and Oseltamivir
Fisher grade for SAH	Fisher IV	Fisher III	Fisher IV	Fisher III and acute IHSDH^∗∗∗^
Neurological symptoms	Anosmia, headache and impaired consciousness	Sudden-onset headache and mental confusion	Impairment of consciousness	Right hemiparesis, aphasia, seizures and impaired consciousness
Hydrocephalus	Yes	No	Yes	No
Days From COVID-19 Symptoms and HSA	Unclear^∗^	4 days	23 days	7 days
SARSCoV-2 involvement	Severe	Mild	Severe	Severe
Digital subtraction angiography (DSA)	Ruptured saccular aneurysm in posterior communicating segment of right ICA	Vasospasm, with no aneurysms or AVMs	Undetermined^∗∗∗∗^	Normal
Treatment for SAH	Nimodipine, anti-vasospasm therapy, EVD^∗∗∗∗∗^ and balloon-assisted embolization of the ruptured aneurysm	Nimodipine, anti-vasospasm therapy	Nimodipine, anti-vasospasm therapy and EVD^∗∗∗∗∗^	Nimodipine, anti-vasospasm therapy
D-dimer	4000 μg/L	1134 μg/L	2850 μg/L	1360 μg/L
CRP	4 mg/L	>9 mg/L	7 mg/L	270 mg/L
Outcome	Discharged to Rehabilitation (mRS: 4)	Discharged Home (mRS: 2)	Death (mRS: 6)	Discharged to Rehabilitation (mRS: 4)

## 5 Discussion

The pandemic caused by the novel coronavirus disease 2019 (COVID-19) has had a profound impact on healthcare systems around the world.^[16,17]^ After several evidences in the literature of a relationship between COVID-19 and cerebrovascular disease, there is an imperative need to define these associations and outcomes. We report the clinical and radiological features of 4 patients confirmed with COVID-19, who developed spontaneous SAH during infection. In 1 case, there was an association with IHSDH.

Most patients were elderly females. All patients had severe symptoms of COVID-19 before SAH. In all cases, chest CT-scan showed bilateral ground-glass opacities. However, according to the current evidences, SAH and intracranial hemorrhages (ICH) have been rarely associated with COVID-19. Mao et al [29] reported in a retrospective case series, neurological manifestations of patients with COVID-19 in China. One out of the 214 patients included in the study were noted to have an ICH. Sharifi et al^[18]^ reported a case of intracranial bleed in a 79-year-old man positive for COVID-19, with no previous history of hypertension or use of anticoagulants. In this case, cerebral CT scan showed a massive bleed within the right hemisphere with intraventricular and subarachnoid extension. Morassi et al^[19]^ also shored 2 cases of severe SARS-CoV-2 infection and acute hemorrhagic stroke. Likewise, Li et al reported in detail, in a cohort of patients positive for COVID-19, 1 case with cerebral venous sinus thrombosis, and 1 with ICH.^[20]^ Poyiadji et al^[21]^ revealed 1 case of acute respiratory symptoms (positive for SARS-CoV-2) with altered mental status and acute hemorrhagic necrotizing encephalitis.

In our case series, patients had neurological symptoms related to SAH an average of 8.75 days after the onset of respiratory symptoms related to COVID-19 infection. All evolved with Fisher III SAH (2 cases cases) and Fisher IV SAH (2 cases). Two cases presented hydrocephalus and required EVD implantation. The possible association and pathophysiology behind the occurrence of SAH in patients with SARS-CoV-2 is still to be determined. There are multiple, not mutually exclusive, and possible mechanisms that may suggest that association. As demonstrated by Tang et al,^[22]^ severe COVID-19 has been related to systemic hyperinflammatory state (cytokine storm) and hyperviscosity. This pro-inflammatory state is associated with vascular injury, including breakdown of collagen and increased permeability of blood-brain barrier (BBB). According to Yeo et al^[23]^ some viruses, such as Influenza A, are able to modulate the expression of matrix metalloproteinase-9 (MMP-9) and matrix metalloproteinase-2 (MMP-2) on the epithelial cell line. Thus, BBB may be disturbed by involvement of systemic elevated MMP-9; that breaks collagen of the arterial walls, in its basal membrane. According to Hackenberg et al^[24]^ elevated turnover rates and instability of arterial collagen are strongly associated with hemodynamic changes and increased risk of SAH, related to intracranial aneurysms. In addition, Qin et al,^[25]^ demonstrated that COVID-19 are capable of induce cytokine storm (hypercytokinemia) leading to elevated systemic inflammation with high levels of mediators, such as IL-1β, IL-6, and TNFα. This may consequently induce vascular injury.^[24,26,27]^

This is a retrospective study and no CRP was performed for SARS-CoV-2 in the CSF. This is a limitation of our research. Therefore, we could not determine precisely the invasion of this virus in the central nervous system. Nevertheless, neurological symptoms such as headache, anosmia, seizures and impairment of consciousness were reported in this study. It is not clear whether the novel coronavirus is neurotropic in humans or whether direct neuroinvasion would be plausibly achieved by infection of vascular endothelium.^[12]^ Ding et al^[28]^ demonstrated systemic vasculitis and vasculitis of cerebral venules at the autopsy in a series of patients with COVID-19. According to Wu et al.^[29]^ SARS-CoV-2 can be found in the brain or CSF. Endothelial damage could explain neurologic symptoms of COVID-19, as well as the propensity for cerebral hemorrhagic events. Angiotensin-converting enzyme 2 (ACE II), highly expressed in lung alveolar type 2 cells, is also present in multiple tissues, including heart, kidneys and cerebrovascular endothelial cells.^[30,31,32]^ Therefore, it would be rational to suggest that ACE II could be involved in COVID-19 neurovascular infection, leading to autoregulation disruption and episodes of high blood pressure spikes, which could result in arterial wall rupture and SHA or ICH. Following the same reasoning, other etiologies of cerebral vasculitis, such as drug abuse, systemic lupus erythematosus, pregnancy or postpartum period and some infectious processes can also lead to intracranial hemorrhage, as previously reported.^[31,33]^

In this series of 4 cases, 3 underwent cerebral angiography, and a ruptured cerebral aneurysm was found in only 1 case. DSA showed absence of cerebral aneurysms or AVMs in 2 cases. In one of these cases, however, signs of diffuse vasospasm were seen. It is known that the main etiology of spontaneous SAH is aneurysmal rupture. According to North American data, non-traumatic SAH occurs in 30,000 patients per year and represents 5% of strokes. Due to its high mortality (approximately 45% in the first 30 days), its etiological identification is imperative.^[34]^ However, the incidence of negative DSA in spontaneous SAH has been reported in the literature, ranging from 2% to 24%.^[35,36]^ In this group of patients, the performance of a new DSA after 3 weeks may not definitively exclude an aneurysm.^[37,38]^ Iwanaga et al^[39]^ demonstrated in a series of patients with SAH and normal initial angiography, that the repetition of this test revealed an aneurysm in 7 of 10 patients with blood predominantly in the anterior interhemispheric fissure and in 1 of 3 patients with blood mainly in the sylvian fissure. In our study, DSA was negative in 2 of 3 tests performed (33.3% of cases). DSA is the gold standard for the diagnosis of vascular lesions resulting in SAH, with a sensitivity of around 99%.^[40,41]^ There are some factors that can lead to failure to identify structural lesions in spontaneous SAH. These include blood in cistern hiding the aneurysm, thrombus inside the aneurysmal sac, proximal and distal vasospasm leading to nonfilling of aneurysm, image impairment by the proximity of skull bone, very small aneurysm, hemorrhage from a venous system and a technically inadequate examination of the posterior circulation.^[42,43]^

In light of the high percentage of negative DSA, the authors of this study raise questions about whether there are different pathophysiological mechanisms that could explain SAH in patients infected with SARS-CoV-2, differently from SAH in the general population. We analyzed the frequency of SAH in non-COVID patients between March and May 2020, compared to the same interval in 2019. Although we observed a 39% reduction in the number of patients hospitalized for SAH, we obtained an increase of 112.5% in the number of non-aneurysmatic SAH. Therefore, in the interval analyzed, we detected 34% of negative DSA compared to the same period in 2019 (only 16%). Thus, we observed a marked change in the presentation of this disease.

In the patients analyzed in this study, we also observed a marked elevation of serum inflammatory markers, such as D-dimer (which ranged from 1134 to 4000 with an average of 2336) and CRP 3835 mg/dl (range 27 −56). Regarding the clinical outcome, we obtained an average mRS of 4, with 1 death, 2 patients with severe functional disability and 1 case with good functional status. Therefore, taking into account that approximately 40% of deaths related to SARS-CoV-2 are related to vascular complications, we should start to consider this disease as a potential systemic vascular infection and not simply an acute respiratory disorder.^[44,45]^ Possibly, a better understanding of this virus and its associated pathophysiological mechanisms will aid in the early diagnosis, treatment, and prevention of SHA and ICH in infected patients.

## 6 Conclusion

In summary, the study illustrates a series of 4 rare cases of SHA in patients infected with the novel Coronavirus. However, cerebral aneurysm was identified in only 1 case. The possible mechanisms underlying the involvement of SARSCoV-2 and SHA are yet to be fully understood. To some extent, the influence of an exacerbated systemic inflammatory process caused by this virus may be one of the possible reasons for SHA in these patients. Therefore, SHA may be included in severe neurological manifestations in patients with COVID-19. However, further epidemiological/clinical studies are needed to confirm the relationship between COVID-19 infection and SAH.

## Author contributions

**Conceptualization:** Auricelio Batista Cezar Junior, Igor Vilela Faquini, Eduardo Vieira de Carvalho Junior, Hetevaldo Tavares Lira Filho, Erton Cesar de Albuquerque Pontes.

**Data curation:** Auricelio Batista Cezar Junior, Luiz Euripedes Almondes Santana Lemos, Joao Batista Monte Freire Filho.

**Formal analysis:** Auricelio Batista Cezar Junior, Jose Laercio Júnior Silva, Eduardo Vieira de Carvalho Junior, Hetevaldo Tavares Lira Filho, Erton Cesar de Albuquerque Pontes, Nivaldo Sena Almeida.

**Investigation:** Auricelio Batista Cezar Junior, Igor Vilela Faquini, Jose Laercio Júnior Silva, Luiz Euripedes Almondes Santana Lemos, Joao Batista Monte Freire Filho, Hetevaldo Tavares Lira Filho.

**Methodology:** Eduardo Vieira de Carvalho Junior, Nivaldo Sena Almeida.

**Project administration:** Auricelio Batista Cezar Junior, Igor Vilela Faquini, Jose Laercio Júnior Silva, Luiz Euripedes Almondes Santana Lemos, Hetevaldo Tavares Lira Filho.

**Resources:** Joao Batista Monte Freire Filho, Erton Cesar de Albuquerque Pontes.

**Software:** Eduardo Vieira de Carvalho Junior.

**Supervision:** Auricelio Batista Cezar Junior, Jose Laercio Júnior Silva, Hildo Cirne Rocha Azevedo-Filho.

**Validation:** Eduardo Vieira de Carvalho Junior, Joao Batista Monte Freire Filho, Nivaldo Sena Almeida.

**Visualization:** Auricelio Batista Cezar Junior, Igor Vilela Faquini, Jose Laercio Júnior Silva, Luiz Euripedes Almondes Santana Lemos, Hildo Cirne Rocha Azevedo-Filho.

**Writing – original draft:** Auricelio Batista Cezar Junior, Jose Laercio Júnior Silva, Joao Batista Monte Freire Filho, Nivaldo Sena Almeida, Hildo Cirne Rocha Azevedo-Filho.

**Writing – review & editing:** Auricelio Batista Cezar Junior, Igor Vilela Faquini, Jose Laercio Júnior Silva, Erton Cesar de Albuquerque Pontes, Hildo Cirne Rocha Azevedo-Filho.
